# Polymorphonuclear Cell Chemotaxis and Suicidal NETosis: Simultaneous Observation Using fMLP, PMA, H7, and Live Cell Imaging

**DOI:** 10.1155/2020/1415947

**Published:** 2020-08-17

**Authors:** Delou Pai, Michael Gruber, Sophie-Marie Pfaehler, Andre Bredthauer, Karla Lehle, Benedikt Trabold

**Affiliations:** ^1^Department of Anesthesiology, University Medical Center Regensburg, Franz-Josef-Strauß-Allee 11, 93053 Regensburg, Germany; ^2^Department of Cardiothoracic Surgery, University Medical Center Regensburg, Franz-Josef-Strauß-Allee 11, 93053 Regensburg, Germany

## Abstract

Chemotaxis and the formation of suicidal neutrophil extracellular traps (suicidal NETosis) are key functions of polymorphonuclear cells (PMNs). Neutrophil extracellular traps in particular are known to be significantly involved in the severity of inflammatory and immunological disorders such as rheumatoid arthritis and Crohn's disease. Therefore, detailed knowledge of PMNs is essential for analyzing the mechanisms involved in, and developing new therapies for, such diseases. To date, no standard method to analyze these cell activities has been established. This study used in vitro live cell imaging to simultaneously observe and analyze PMN functions. To demonstrate this, the effects of phorbol-12-myristat-13-acetat (PMA, 0.1-10 nM), N-formylmethionine-leucyl-phenylalanine (fMLP, 10 nM), and protein kinase C inhibitor 1-(5-isoquinolinesulfonyl)-2-methylpiperazine (H7) on PMN chemotaxis and suicidal NETosis were studied. PMA (1 nM-10 nM) resulted in significant concentration-dependent behavior in chemotaxis and an earlier onset of maximum oxidative burst and NET formation of up to 44%. When adding H7, PMA-triggered PMN functions were reduced, demonstrating that all three functions rely mostly on protein kinase C (PKC) activity, while PKC is not essential for fMLP-induced PMN activity. Thus, the method here described can be used to objectively quantify PMN functions and, especially through the regulation of the PKC pathway, could be useful in further clinical studies of immunological disorders.

## 1. Introduction

Neutrophils, also known as polymorphonuclear cells (PMNs), constitute 50–60% of human leukocytes [1]. They are formed in the bone marrow and, once matured, enter into the blood stream [2] and swiftly migrate through tissue to detect low concentrations of chemoattractants. These substances enable PMNs to be the first cells to arrive at the site of infection and destroy any potential pathogens by means of phagocytosis, degranulation, cytokine production, and NETosis. Therefore, PMNs are of great importance to the immune system [3].

NETosis describes a mechanism by which a neutrophil chromatin remodels into a net-like structure and is then ejected into the extracellular space to form a neutrophil extracellular trap (NET). To date, several types of NETosis have been identified, such as suicidal [4], vital [5], and a variation of the suicidal type based on mitochondrial DNA [6]. The NET consists of neutrophil DNA, histones, and proteins, enabling the NET to bind and kill pathogens [7]. Apart from preventing infections, NETs also play an important role in immunological disorders such as systemic lupus erythematodes, Crohn's disease, ulcerative colitis, multiple sclerosis, and rheumatoid arthritis (RA) as well as diabetes types 1 and 2. In most cases, the cause is either a lack of or excessive NET formation [2]. Consequently, detailed knowledge of how PMNs work is a key to understanding and curing a wide range of diseases.

PMN plays a crucial role in the onset and progression of RA. In RA, PMN displays increased cell survival, migration, and inflammatory activity and enhanced NET formation, leading to the activation of other immune cells [8]. Upon entering the synovial cavity, rheumatoid nodules, or skin [9], enhanced NET formation sets in. Among other processes, the intracellular peptidylarginine deiminase 4 citrullinates several peptides present on the NET before extrusion [10]. Together with proinflammatory cytokines such as IL17A and TNF [2], these citrullinated peptides act as extracellular autoantigens. Fibroblast-like synoviocytes, which serve as antigen-presenting cells, internalize the citrullinated peptides and induce the formation of anti-citrullinated protein antibodies (ACPAs) [10]. After antigen recognition by ACPAs, immune complexes form, which in turn induce further NET formation. A continuous cycle is established, leading to joint destruction. ACPA levels are elevated in RA patients before clinical symptoms can be observed, thus serving as an early biomarker. In addition, fibroblast-like synoviocytes increase the synthesis of adhesion molecules and inflammatory cytokines such as IL-6 or IL-8, contributing to pathogenic autoimmunity and further damage to tissue and joints [9]. Aside from NETs, several other substances are released into the synovia, such as B-cell activating factor and nuclear factor kappa-b ligand, leading to excessive immune responses in joints and tissue due to the increased activity of B-cells and osteoclasts [2]. Therefore, targeting neutrophil-produced cytokines, chemokines, and NET formation to regulate PMN activity offers a novel therapy concept towards the improvement of RA outcomes [11].

To observe PMN functions in vitro, several chemotactic substances are used, such as pathogen-derived N-formyl-methionine-leucyl-phenylalanine (fMLP) or host-derived IL-8 [12, 13]. fMLP, for instance, modulates PMN adhesion [14], microtubule polymerization [15], and activation of protein kinase C (PKC) isoforms [16] via its receptor-associated tyrosine kinases [17]. The PKC isoforms are a group of enzymes involved in several PMN functions. They can be activated indirectly (fMLP) and directly through pharmacological agents such as phorbol-12-myristat-13-acetat (PMA). After activation, PKC translocate to cell compartments such as the cell membrane and are identifiable through immunochemical studies. Five of the many PKC isoforms have been identified in PMN so far: *α*, *β*1, *β*2, *δ*, and *ζ*. Each of these isoforms contributes differently to PMN functions. PKC*β*, for example, regulates ROS production [18], whereas PKC*ζ* contributes to both PMN migratory [19] and oxidative behavior [20].

In experimental trials, no gold standard in vitro method to analyze PMN functions such as NETosis has been found yet. One of the most common methods in PMN research involves the isolation of PMNs by density gradient, incubation with a stimulus of choice, then fixation followed by immunostaining, and lastly examination through microscopy. This process enables a detailed study of the structures involved, depending on the immunostaining. However, as PMNs are no longer viable after fixation, the resulting analysis depicts only a single point in time, and in most cases, this analysis is subject to the examiner's opinion [4].

In addition, flow cytometry is often used to investigate cell activity. Other methods, such as fluorometry or ELISA, are more applicable in a clinical setting and, similar to microscopy, can identify NET components. The drawback is that neither method is able to determine the origin of the component, and the amount of the component is not measured over time but accumulated up to the point of detection [21].

Each of the methods stated here has its advantages and disadvantages, meaning it is up to the researchers to decide which method is the most suitable for their investigation. Live cell imaging [12, 22] uses viable neutrophils with the possibility of cell observation over any length of time and using any desired stimulus. With further development, this method could become the new gold standard, combining its advantages with solutions to the disadvantages of former methods.

This study is aimed at

1. demonstrating a method that enables the objective and simultaneous observation of motility and cell activities of viable PMNs over a longer period of time by using live cell imaging
2. delivering quantitative results on the effects of fMLP, PMA, and H7 on PMN migration, oxidative activity, and formation of neutrophil extracellular traps to further understand PMN behavior and provide insight for potential clinical application

## 2. Material and Methods

### 2.1. PMN Isolation

Peripheral blood was drawn from healthy donors using Safety-Multifly-cannula (0.8 × 19 mm), safety-cannula (0.9 × 38 mm), and S-Monovettes containing lithium heparin (Li-Hep (16 IE/mL blood); all equipment above is from Sarstedt AG & Co, Nümbrecht, Germany) according to a protocol approved by the ethics committee (No.15-101-0043) at the University of Regensburg (Germany). PMNs were isolated using density gradient centrifugation based on the protocol provided by pluriSelect Life Science using LeukoSpin and LymphoSpin solutions (pluriSelect Life Science, Leipzig, Germany) [23]. The cells were then resuspended in RPMI 1640 medium (Sigma Aldrich, Steinheim, Germany) at a concentration of 18 × 10^6^ cells/mL. PMNs were stained using 4′,6-diamidino-2-phenylindole (DAPI, Sigma Aldrich Chemie Ltd., St. Louis, USA) to mark extracellular DNA and dihydrorhodamine-123 (DHR-123, Molecular Probes, Inc., Eugene, USA) to detect reactive oxygen species (ROS). Both markers were initially diluted with Dimethylformamide (DMF) and Dulbecco's phosphate-buffered saline (DPBS, both from Sigma Aldrich, Steinheim, Germany), followed by RPMI 1640 medium with 10% fetal calf serum (FCS, Sigma Aldrich, Steinheim, Germany) to reach their final concentrations of 50 ng/mL and 10 nM, respectively.

### 2.2. Stimulation

All stimulants and isoquinoline-5-sulfon-2-methyl-1-piperazide (H7, Sigma Aldrich, Steinheim, Germany) in a hydrochloride solution (Honeywell Fluka, Seelze, Germany) were diluted using RPMI 1640 medium with 10% FCS. N-Formyl-methionine-leucyl-phenylalanine (fMLP, Sigma Aldrich Chemie Ltd., St. Louis, USA) was diluted to its final concentration of 10 nM, whereas H7 was applied at final concentrations between 25 *μ*M and 8 mM. Phorbol-12-myristat-13-acetat (PMA, Sigma Aldrich Chemie Ltd., St. Louis, USA) was used at final concentrations of 0.1, 1, 3, 10, 80, and 100 nM.

### 2.3. IBIDI-Slide Preparation

24 hours prior to each observation, the *μ*-Slide chemotaxis (IBIDI-Slide, IBIDI Ltd., Planegg, Germany) was stored in an incubator at 37°C and 5% CO_2_. Each IBIDI-Slide consists of three chambers, with each chamber consisting of a central channel and a reservoir on each side. Cell medium was prepared in advance using 60 *μ*L Minimum Essential Media (MEM, PAN-Biotech Ltd., Aidenbach, Germany), 60 *μ*L H_2_O, 30 *μ*L sodium hydrogen carbonate (1 M, NaHCO_3_, VWR International Ltd., Radnor, Pennsylvania, USA), and 150 *μ*L RPMI 1640 and kept in the incubator next to the IBIDI-Slide. The PureCol Type I Bovine Collagen Solution (collagen, 3 mg/mL, Advanced BioMatrix Carlsbad, USA) was at room temperature when used. H7 was added to the PMN solution and incubated for 15 minutes before the addition of other solutions. 50 *μ*L of the prepared PMN solution, 100 *μ*L of cell medium, 30 *μ*L PMA, and 150 *μ*L collagen were combined. 7 *μ*L of this mixture was used to fill the central channels of an IBIDI-Slide. Each channel contained a different concentration of PMA, with one channel serving as a control. The IBIDI-Slide was then incubated for 30 minutes at 37°C and 5% CO_2_. Afterward, the right reservoir of each chamber was filled with 65 *μ*L of RPMI 1640 and 10% FCS, whereas the left reservoir contained 65 *μ*L of fMLP (10 nM in RPMI/10% FCS). The IBIDI-Slide was then sealed and inserted into a Leica DMi8 microscope (Leica Mikroskopie & Systeme Ltd., Wetzlar, Germany).

### 2.4. Microscopy

Live cell imaging was done at 30-second time intervals for a total duration of 4.5 hours using a Leica DFC9000 GT camera (Leica Mikroskopie & Systeme Ltd., Wetzlar, Germany), a CoolLEDpE4000 light source (CoolLED Ltd., Andover, Great Britain), and Leica Application Suite X Software (LAS X 3,0,4,16529, Leica Mikroskopie & Systeme Ltd., Wetzlar, Germany). A dual filter cube (Chroma Technology Corp., Vermont, USA) with the following characteristics was used: excitation windows between 380-410 nm and 472-498 nm, dichroic filters at 418 nm and 502 nm, and emission windows between 424-460 nm and 505-545 nm.

### 2.5. Image Analysis

Images were analyzed using IMARIS 9.02 Software (Bitplane, Zurich, Switzerland). The IMARIS “Surface” tool measured ROS production and NET formation according to the changes in rhodamine-123 and DAPI fluorescence, respectively. The development of ROS production was quantified by calculating the time of maximal ROS production (*t*_max_ROS) in minutes. NET formation is described as E_T50_NETs in minutes, which is the time point of half-maximum effect of NET formation reached during the experiment. The sufficiency of analyzing time-resolved DAPI staining for NETosis identification has already been proven [22, 24]. The “Spots” tool tracked each cell movement for the entire period of observation. Track length, track displacement along the *x*-axis and *y*-axis (TDX, TDY), and the directness were measured to describe migratory behavior. *D* describes migration efficiency and takes values between 0 and 1. The 1 value corresponds to straight migration as the Euclidean distance matches the accumulated track length, whereas a value close to 0 is equal to a migration in which the track length is far greater than the Euclidean distance. All migration parameters were grouped in 30-minute intervals before statistical analysis.

### 2.6. Statistical Analysis

IBM SPSS Statistics 22 Software was used for statistical analysis. Data are presented as means ± SEM or median with confidence interval, depending on the distribution. When normal distribution was present, parametric tests (Student *t*-test, post hoc analysis) were applied, whereas nonparametric tests (Mann-Whitney *U* test, Kruskal-Wallis test) were applied when normal distribution could not be achieved despite data transformation. The Pearson correlation was conducted once significance was proven. *P* values below 0.05 were considered significant if not stated otherwise.

## 3. Results

### 3.1. Chemotaxis Is Dependent on PMA Concentration

**Figure 1:**
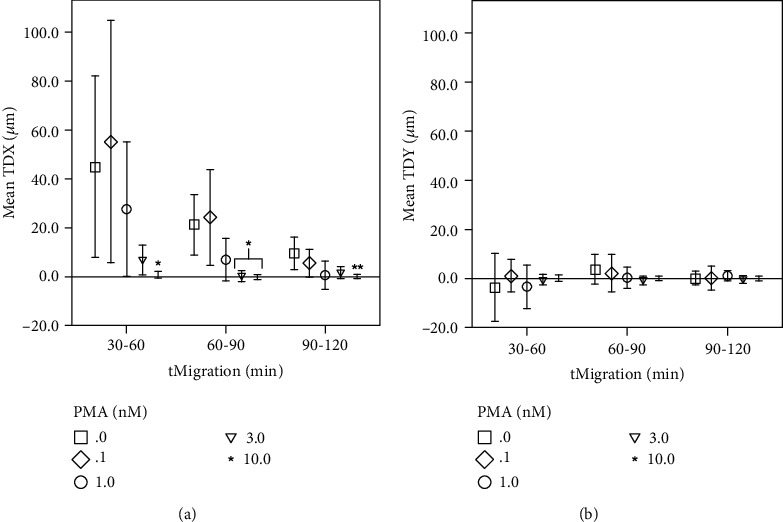
Track displacement decreases during time-lapse and PMA. *x*-axis: 30-minute time intervals tMigration (min) in minutes, *y*-axis: (a) mean track displacement along the *x*-axis TDX in *μ*m; (b) mean track displacement along the *y*-axis TDY (*μ*m) in *μ*m; comparing different PMA concentrations (0–10 nM); ^∗^significant when compared to 0 nM and 0.1 nM PMA; ^∗∗^significant when compared to 0 nM PMA; error bars represent standard deviation of measurements.

TDX decreased significantly from 44.8 *μ*m (SD 44.6 *μ*m, *n* = 8, 0 nM PMA) to 0.6 *μ*m (SD 2.13 *μ*m, *n* = 14, 10 nM PMA) over 30–60 minutes of observation in the presence of PMA (see Figure 1(a)). TDY, by contrast, fluctuated at the zero value with and without PMA (see Figure 1(b)).

**Figure 2:**
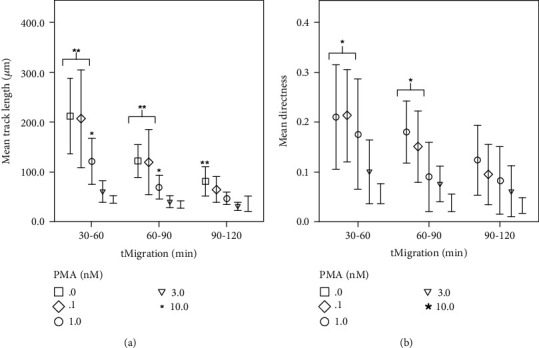
Track length and directness decrease during time-lapse and PMA. *x*-axis: 30-minute time intervals tMigration (min), *y*-axis: (a) mean track length (*μ*m) in *μ*m, comparing different PMA concentrations (0–10 nM); (b) mean directness, comparing different PMA concentrations (0–10 nM); ^∗^significant when compared to 10 nM PMA; ^∗∗^significant when compared to 3 nM and 10 nM PMA; error bars represent standard deviation of measurements.

The same concentration-dependent behavior was seen with a track length decrease of 79.2% from 211.7 *μ*m (SD 90.6 *μ*m, *n* = 8, 0 nM PMA) to 44.1 *μ*m (SD 12.7 *μ*m, *n* = 14, 10 nM PMA) during 30–60 minutes of observation in the presence of PMA (see Figure 2(a)). When comparing PMA concentrations below 1 nM (*n* = 19) and those higher than 3 nM PMA (*n* = 26), a significant decrease in track length (see Figure 2(a)) was observed for each time frame measured, ranging from 79.2 to 55.8% (10 nM PMA) and 62.0 to 71.4% (3 nM PMA). There was a similar significant decrease in directness by 73.3–71.4% (see Figure 2(b)) during 30–90 minutes of observation when comparing 10 nM (*n* = 14) and 0.1 nM (*n* = 11) PMA. Once chemotaxis ceased, increased ROS production inside the PMNs was observed in the video footage. During this process, morphological changes took place, followed by a rupture of the cell membranes, thus extruding the PMNs' DNA into the extracellular space.

### 3.2. Time of Maximal ROS Production and NET Formation Are Dependent on PMA Concentration

**Figure 3:**
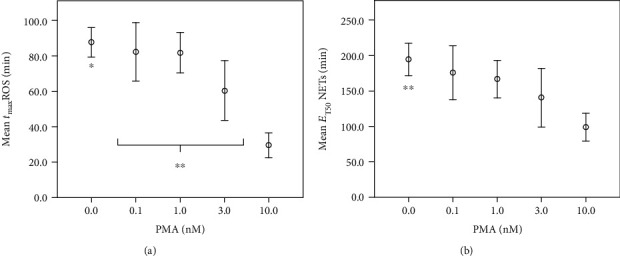
Point of time of ROS production and half-maximal NET formation decrease during PMA exposition. *x*-axis: PMA (nM) concentration in nM; *y*-axis: (a) mean time of maximal ROS formation *t*_max_ROS (min) in min; (b) mean time of half-maximal NET formation E_T50_NETs in minutes; ^∗^significant compared to 3 nM and 10 nM PMA; ^∗∗^significant (*P* < 0.05) compared to 10 nM PMA; error bars represent standard deviation of measurements.

*t*_max_ROS decreased by up to 64.3% (10 nM PMA, *n* = 14) in a PMA concentration-dependent manner. Comparisons of 10 nM with none, 0.1, or 1 nM PMA (*P* < 0.001) and comparisons of 3 nM (*n* = 12) and 0 nM (*n* = 17) or 10 nM PMA (*n* = 14) showed a significant impact on *t*_max_ROS (see Figure 3(a)). E_T50_NETs (see Figure 3(b)) decreased significantly (*n* = 13) by a mean value of 85.2 minutes with 10 nM PMA stimulation compared to the control. The Pearson correlation was significant (*P* < 0.001) for PMA-dependent decreases of *t*_max_ROS and E_T50_NETs parameters with negative coefficients at -0.745 and -0.523, respectively.

### 3.3. H7 Inhibits fMLP- and PMA-Induced Chemotaxis

**Figure 4:**
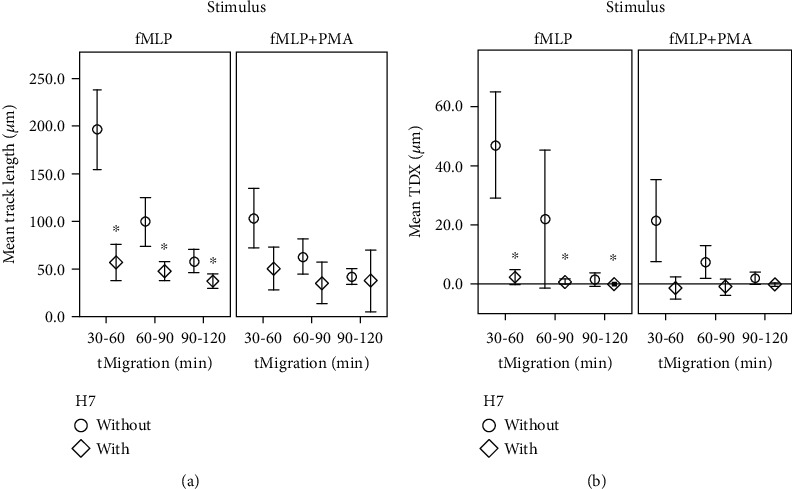
Chemotaxis decreases during H7 exposition. *x*-axis: 30-minute time intervals tMigration (min), *y*-axis: (a) mean track length in *μ*m; (b) mean track displacement along the *x*-axis TDX in *μ*m; comparing groups with and without H7; ^∗^significant (*P* < 0.05) compared to the control group without H7; error bars represent standard deviation of measurements.

PKC inhibitor H7 (*n* = 17) significantly decreased fMLP-induced migration (see Figure 4) in track length, TDX, and directness, nearly halting any observable movement.

Track length decreased by a mean value of 35.6-68.7%, depending on the time frame observed. However, the addition of H7 to fMLP- and PMA-induced chemotaxis (*n* = 5, see Figure 4) did not show any significant effect when compared to the control group (*n* = 14).

### 3.4. H7 Inhibits PMA but Not fMLP-Induced ROS Production or NET Forming

**Figure 5:**
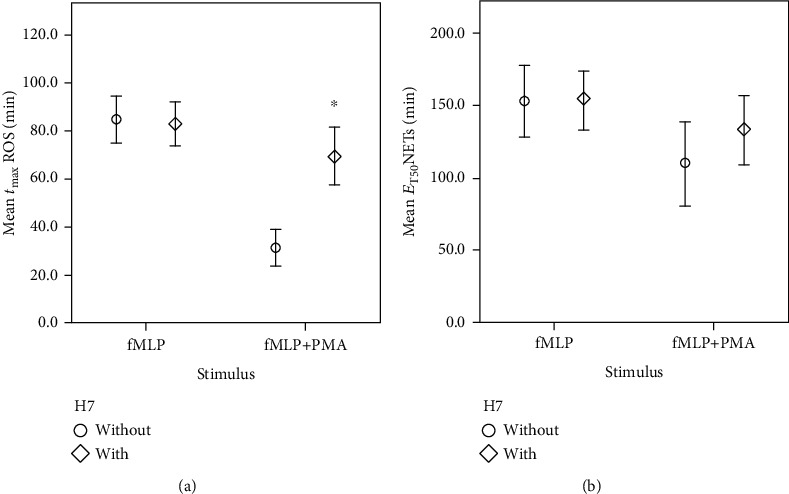
Delayed onset of oxidative activity and NET formation triggered by fMLP+PMA during H7 exposition. *x*-axis: cell groups stimulated using fMLP or fMLP and PMA; *y*-axis: (a) mean time of maximal ROS formation *t*_max_ROS in min; (b) mean time of half-maximal NET formation E_T50_NETs in min; ^∗^significant (*P* ≤ 0.001) compared to the control group without H7; error bars represent standard deviation of measurements.

H7 did not have any significant impact on fMLP (*n* = 15)-induced *t*_max_ROS or E_T50_NETs, whereas PMA-induced *t*_max_ROS (*P* < 0.001) and E_T50_NETs were pushed back by mean values of 38.2 minutes (*n* = 5) and 23.3 minutes (*n* = 5), respectively, when adding H7 in this setting (see Figure 5).

### 3.5. PMNs Show a Specific Stimulus-Dependent Time Sequence

**Figure 6:**
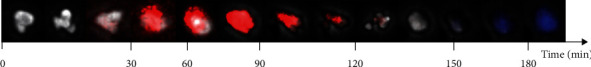
Live cell imaging: time flow in min of a PMN treated with fMLP and H7 showing chemotaxis (phase-contrast), oxidative activity (red, rhodamine-123, staining reactive oxygen species (ROS)), and suicidal NETosis (blue, DAPI, staining PMN–DNA (NET)).

The following sequence of cellular behavior was observed in all test groups, with differences only in the duration of each phase (see Figure 6): in the first part of the phase contrast footage, PMN migration along the fMLP gradient was detected. ROS production was initiated, and by the time the PMNs stop migrating, ROS formation had reached its peak, indicated by increased rhodamine-123 production. The then nonmigrating PMNs underwent morphological changes. The cell nuclei swelled, and the membranes burst, setting the NET free. This process was made visible through DAPI intercalation.

## 4. Discussion

The results reported in this study were achieved by applying a method involving live cell imaging. This cell set-up allows the investigator to vary direct stimuli as well as directional chemoattraction in any manner desired and to analyze these objectively using a single set-up. This is a great advantage when compared to earlier studies which solely involved flow cytometry to analyze PMN behavior [21]. In addition, simultaneous observation enables a comparison of each activity in relation to each other. Doblinger et al. used the same method described here while adding tagged MPO antibodies as an additional marker to stain NETs. As both DAPI and MPO were simultaneously observed and colocated, it was proven that this method is sufficient to observe NETosis. Therefore, only DAPI staining was used in this study to analyze NET formation [22].

Being able to study important PMN functions such as migration and NETosis simultaneously can provide insight into the complex cell mechanisms involved, as will be discussed in the following.

### 4.1. Chemotaxis in the Presence of fMLP, PMA, and Protein Kinase C Inhibitor H7

This study confirms typical fMLP-induced migratory behavior [25]. The method's set-up guarantees a sufficient separation of the two reservoirs through the middle channel filled with a PMN-infused collagen matrix. With one of the two separate reservoirs containing only medium/FCS as a control, the investigator is able to assess the ability of fMLP as a chemoattractive substance through observation of directional migration to one side of the chamber. This is a great advantage over the Dunn chamber assay [3], in which cells are fully surrounded by a chemoattractant gradient at all times. In this study, PMNs clearly showed a migration along the positive *x*-axis towards the fMLP reservoir without major diversion along the *y*-axis. Moonen et al. observed a similar fMLP-directed migration using 15 nM fMLP with PBS as controls in Insall chambers. Values for migration directness, however, differed slightly during the first period of observation [26]. This can be explained by the differences in fMLP concentration and the time frame observed. Adding the PKC inhibitor H7 to fMLP-stimulated PMNs led to a decrease in most migration parameters. The results suggest that PKC plays an important role in fMLP-induced chemotaxis, which coincides with previous findings [27, 28]. Paclet et al. used Ro-31-8220 as a PKC inhibitor and observed a decrease in fMLP-induced (10 nM and 10 *μ*M) chemotaxis as well [29]. Despite using different PKC inhibitors, and taking into consideration the studies stated above, PKC seems to be imperative for fMLP-induced migration.

Surprisingly, H7 had hardly any effect on PMN migration in the presence of PMA, which contradicts previous observations [27]. Assuming that H7 and PMA are not restricted in their functions, two theories could explain this observation. Firstly, the H7 concentrations used were too low, and secondly, the migration observed was already at its minimum upon PMA exposure without adding H7. The latter being the case, no change would be seen when H7 was added to the exhausted PMN. With regard to all migration parameters, this second theory is considered the more likely explanation. Thus, fMLP- and/or PMA-triggered migration is dependent on PKC and can be regulated using the PKC inhibitor H7. There are also other substances capable of modulating PMN migration. Applying the same method described in this paper, PMN migration could either be decreased or increased using substances such as therapeutic anticoagulants [24] or IL-8 [12], respectively. This highlights the advantage of this method's approach: several conditions can be observed simultaneously for comparison.

### 4.2. Oxidative Activity and NET in the Presence of fMLP, PMA, and H7

Once migration came to a halt, oxidative activity sets in. When exposed solely to fMLP (10 nM), *t*_max_ROS peaked at 90 minutes. This suggests that PMN migration and NETosis were triggered by fMLP (10 nM) alone.

Briheim et al. used 100 nM fMLP in their experiments, which resulted in peaks at two and ten minutes. This difference in results can be explained by the differences in methods and concentration of fMLP used. Briheim et al. were observing the development of ROS chemiluminescence [30], whereas the area of ROS producing PMNs over time was observed in this study.

H7 showed no effect on fMLP-induced ROS formation or NETosis. This coincides with a study by Klink et al., in which H7 (100 *μ*M) did not have any impact on ROS formation triggered by fMLP in fluorometry [17]. However, previous studies have also shown that fMLP is in fact involved in PKC*α*, *β*, and *ζ* [20] translocation leading to ROS production. This suggests that either H7 is incapable of inhibiting fMLP-induced PKC activity or fMLP can also activate ROS production in a PKC-independent way. The latter seems more likely because fMLP is closely associated with G-protein coupled pathways, which can lead to ROS production independent of direct PKC activation [31]. In addition, H7 showed an effect on ROS formation and NETosis when the direct PKC activator PMA was present. Kilpatrick et al. attribute the PKC*δ* as playing an initiating role for PMA-induced but not fMLP-induced ROS formation [32]. Other studies state that PKC*α* and PKC*β* are essential for both fMLP- and PMA-associated ROS formation [16]. This makes it less likely that H7 is incapable of inhibiting fMLP-induced PKC activity. We therefore suggest that fMLP-triggered PMN functions are linked to PKC isotypes but are able to coopt alternative fMLP-sensitive signal pathways for ROS formation. This does not apply to PMA-triggered oxidative activity as this has been shown to be susceptible to H7 [33].

Regarding NETosis, E_T50_NETs did not prove to be affected by H7 in a statistically significant way. However, when comparing the E_T50_NETs values in the H7 group and the control group, there was a noticeable difference. Taking into consideration the steady correlation of ROS formation and NET formation in the first series of experiments, and the work of Alemán et al. stating that the inhibition of PKC also delays NET formation [34], a correlation between PMA-induced NET formation and H7 seems likely. From a clinical point of view, it can be suggested that reducing PKC activity in PMN could be of therapeutic use in containing immunological disorders such as rheumatoid arthritis. Otherwise, elevated PMN activity can lead to excessive oxidative activity and NET formation, thus causing further joint deterioration [35].

### 4.3. Concentration-Dependent Effect of PMA on Chemotaxis

In this study, we also analyzed the concentration-dependent effect of PMA on PMN functions. Here, migration showed a decrease during PMA exposure above 1 nM. This observation leads to two hypotheses: firstly, PMA inhibits migration, and secondly, it elevates cell activation. Elevated cell activity would result in an early onset of migration, oxidative activity, and NET formation in accordance with the stimulus used. An early study by Boonen et al. showed a concentration-dependent increase of migration using 0–0.1 nM PMA. This suggests an elevated migratory activity, after stimulation with PMA in low concentrations, similar to the findings in this study (see TDX development in Figure 1). Higher concentrations were thought to be inhibitory to migration [36], supporting the first theory. However, bearing in mind the constant sequence of PMN behavior across all test groups, an inhibition of migration seems highly unlikely. Instead, it is here considered that PMA-triggered elevated cell activation was the cause of an early and intense migration followed by the early onset of oxidative activity and NET formation observed in this study.

### 4.4. Concentration-Dependent Effect of PMA on ROS Production and NET Formation

PMA-induced oxidative activity *t*_max_ROS peaked between 60 and 120 minutes after PMA exposure in a concentration-dependent manner. Looking at PMA concentrations from 1 nM to 10 nM, it was seen that a threefold concentration led to an earlier onset of *t*_max_ROS by 25-30 minutes. These results suggest a linear correlation between PMA concentration and *t*_max_ROS at a low concentration range. Other studies have shown a similar peak time for oxidative burst when using a much higher concentration of PMA (50 nM [37], 100 nM [30]). Two hypotheses can be deduced: Firstly, *t*_max_ROS is dependent on PMA concentration, but probably not in a linear manner. Secondly, PMA-triggered PMN functions show a ceiling effect: PMN activation is already saturated and has reached its maximum when exposed to lower PMA concentrations (e.g., 50 nM), thus triggering intracellular activities which take at least 20 minutes to show maximal effect. This could explain why different studies state similar maximal oxidative activity peak times while using highly different PMA concentrations.

It should be kept in mind that PMA concentrations below 1 nM did not show any significant effect on PMN oxidative activity compared to the control group. In this study, a minimum stimulus of 1-3 nM PMA for at least 30 minutes seemed to be necessary to trigger the intracellular cascade. However, Bonnekoh et al. observed an increased number of NETs in patients with Schnitzler's syndrome, an autoimmune disorder, when using a much lower concentration of PMA (0.05 nM) for 130 minutes [38]. This suggests that much lower concentrations could be sufficient to trigger high NET formation in patients with autoimmune disorders, thus rendering them susceptible to exacerbation from low stimuli. This could explain increased rates and acceleration of NETosis in autoimmune disorders such as rheumatoid arthritis [9] and psoriasis [39].

Furthermore, live cell imaging shows that ROS formation has to take place before the NET is extruded. This is in accordance with the work of Kenny et al. [37]. In addition, both *t*_max_ROS and E_T50_NETs show PMA-dependent behavior, which a Pearson correlation confirms, with *t*_max_ROS being distinctly more sensitive to changes in PMA concentration compared to E_T50_NETs.

Therefore, the concentration and duration of the stimulation and observation have to be taken into consideration when assessing stimulus-dependent PMN activity, especially in a clinical setting. Further evaluation as described in this study of PMN activities in vitro, using human blood of the healthy and the diseased, is needed. This is necessary in order to develop new therapy concepts for immunological disorders.

## 5. Conclusion

PMN migration, ROS production, and NET formation are triggered by chemotactic substances such as fMLP (10 nM). This behavior can be influenced by PMA. During PMA exposure (0.1–10 nM), PMN activity levels increase in a concentration-dependent manner, leading to an early onset of *t*_max_ROS and E_T50_NETs.

All three PMN functions observed are dependent on the PKC activity in the presence of PMA. In the absence of PMA, PKC dependency also applies to fMLP-triggered migration, but not fMLP-triggered ROS formation or NET formation.

The regulation of PKC activity in patients with immunological diseases such as rheumatoid arthritis could offer a new therapeutic concept to contain the disease.

In addition, using live cell imaging as described in this paper allows the investigator to vary stimuli and chemoattractants in any manner desired, thus providing insight into complex cell mechanisms in PMN. The investigation of PMN involvement in various diseases, as well as in pharmacological testing, is feasible by means of the live cell imaging described in this study. Using LASX and Imaris Software allows objective data acquisition and analysis. We believe this approach to be suitable for future PMN research in immunological disorders.

## Data Availability

The datasets generated during and/or analyzed during the current study are available from the corresponding author upon reasonable request.
